# Korean Ginseng Berry Polysaccharide Enhances Immunomodulation Activities of Peritoneal Macrophages in Mice with Cyclophosphamide-Induced Immunosuppression

**DOI:** 10.4014/jmb.2211.11056

**Published:** 2023-03-30

**Authors:** JeongUn Choi, Ju Hyun Nam, Weerawan Rod-in, Chaiwat Monmai, A-yeong Jang, SangGuan You, Woo Jung Park

**Affiliations:** 1 Department of Wellness-Bio Industry, Gangneung-Wonju National University, Gangneung, Gangwon 25457, Republic of Korea; 2 Department of Marine Food Science and Technology, Gangneung-Wonju National University, Gangneung, Gangwon 25457, Republic of Korea

**Keywords:** Immune system, ginseng berry, polysaccharides, macrophages

## Abstract

Korean ginseng (*Panax ginseng* C. A. Meyer), a member of the Araliaceae family, is known as a traditional medicinal plant to have a wide range of health properties. Polysaccharides constitute a major component of Korean ginseng, and its berries exhibit immune-modulating properties. The purpose of this study was to investigate the immune effects of crude polysaccharide (GBPC) extracted from Korean ginseng berry on peritoneal macrophages in mice with cyclophosphamide (CY)- induced immunosuppression. BALB/c mice were divided into eight groups: normal control, normal control + CY, levamisole + CY, ginseng + CY, and four concentrations of 50, 100, 250, and 500mg/kg BW/day of GBPC + CY. Mice were orally administered with samples for 10 days. Immunosuppression was established by treating mice with CY (80 mg/kg BW/day) through intraperitoneal injection on days 4 to 6. The immune function of peritoneal macrophages was then evaluated. Oral administration of 500mg/kg BW/day GBPC resulted in proliferation, NO production, and phagocytosis at 100%, 88%, and 91%, respectively, close to the levels of the normal group (100%) of peritoneal macrophages. In CY-treated mice, GBPC of 50−500 mg/kg BW/day also dose-dependently stimulated the proliferation, NO production, and phagocytosis at 56−100%, 47−88%, and 53−91%, respectively, with expression levels of immune-associated genes, such as *iNOS*, *COX−2*, *IL−1β*, *IL−6*, and *TNF–α*, of about 0.32 to 2.87-fold, compared to those in the CY group. GBPC could be a potential immunomodulatory material to control peritoneal macrophages under an immunosuppressive condition.

## Introduction

Macrophages can release pro-inflammatory cytokines, such as tumor necrosis factor (TNF)–α, interleukin (IL)−6, and IL−1β, as well as mediators that induce inflammation, such as nitric oxide (NO) and prostaglandin E_2_ (PGE_2_), which have significant functions through both innate and adaptive immunity [1, 2]. In mice, peritoneal macrophages have been widely used as tissue macrophage compartments, because the peritoneal cavity provides a convenient location for extracting a large number of resident macrophages [3]. A variety of effector mechanisms and immune systems responsible for the regulation permit them to play an important role in controlling infectious and inflammatory diseases [3, 4].

Cyclophosphamide (CY) is one of the most effective anticancer drugs used in chemotherapy [5]. It is an inactive prodrug that requires enzymes and chemicals to form DNA crosslinks, which gives it its cytotoxic properties, to treat certain types of cancer, including breast cancer, lymphoma, and pediatric tumors [6]. However, it has a number of adverse reactions, such as immunosuppression, cytotoxicity, oxidative stress, leukopenia, and myelosuppression [7, 8]. CY has been widely used to develop animal models with immunosuppressive effects for many natural substances, and they are known to activate cells involved in immune function, such as lymphocytes, macrophages, and natural killer (NK) cells [2, 9-11].

Natural macromolecular polymers known as polysaccharides are often made up of long chains of monosaccharides connected by glycosidic linkages in linear or branching chains [12]. Generally, plant-derived polysaccharides can act as an immunomodulatory therapeutic agent by stimulating macrophages to create NO, cytokines, chemokines, and reactive oxygen species (ROS), as well as cytotoxic activity [8, 13-16]. Previous research has revealed the immunomodulatory activity of polysaccharides derived from plants such as *Hippophae rhamnoides* [2], *Ganoderma atrum* [11], *Craterellus cornucopioides* [9], *Dioscorea opposite* [17], *Pleurotus nebrodensis* [18], *Panax ginseng* [19, 20], and *P. quinquefolius* [21] in a murine model of immunosuppression induced by CY.

Korean ginseng (*Panax ginseng* C. A. Meyer, Araliaceae) is one of the most popular food and medicine plants in East Asian countries, such as Korea, China, and Japan [22, 23]. The main active ingredients of Korean ginseng include ginsenosides, saponin, polysaccharides, amino acids, fatty acids, nitrogen-containing compounds, and phenolic compounds [23]. The leaves and fruits of *P. ginseng* also show pharmacological activities similar to roots [24]. In particular, ginseng polysaccharides have been demonstrated to possess various pharmacological activities, such as immunomodulation [14, 25], antitumor [25, 26], antimicrobial [27], antioxidant [28, 29], anti-hyperglycemic [29], and anti-adhesive [30] effects. Polysaccharides of *P. ginseng* also exhibit immunomodulatory effects by stimulating the immune response functions of splenic lymphocytes, mouse peritoneal macrophages, and other immune cells in biological studies in vitro and in vivo [19, 24, 31].

Using gel filtration chromatography, Ginseng berry polysaccharide fraction has been revealed substantially boost TNF–α, IL−6, and IL−12 production in mice peritoneal macrophages [31]. One polysaccharide isolated from ginseng fruits can reduce tumor growth and encourage immunological function in Lewis lung carcinoma (LLC)-bearing mice [24]. Recently, a crude polysaccharide extracted from Korean ginseng berries (GBPC) with molecular weights of 328.4 and 54.2 kDa was discovered to be mainly composed of galactose, rhamnose, glucose, mannose, and arabinose [32]. Our previous study demonstrated that GBPC possessed immune-enchaining properties in RAW 264.7 macrophages and splenic lymphocytes under immunosuppression caused by CY treatment [32, 33], but the underlying biomarker in peritoneal macrophages to enchain immunity remains unclear. Thus, the current investigation was aimed at identifying the immunomodulatory effects of crude polysaccharides isolated from Korean ginseng berries on mouse peritoneal macrophages using mice with immunosuppression induced by CY.

## Materials and Methods

### Extraction of Polysaccharide

Crude polysaccharides (GBPC) were extracted from Korean ginseng berries, as obtained in our previous report [32], which reported the monosaccharide contents of GBPC to be composed of total carbohydrate, sulfate, uronic acid, and protein of (85.4, 5.5, 1.2, and 11.3)%, respectively.

### Reagents and Materials

Cyclophosphamide (CY), saline solution, levamisole, lipopolysaccharide (LPS), Griess reagent, and neutral red solution, were provided by Sigma–Aldrich (USA). Commercial red ginseng syrup was purchased from the Korea Ginseng Corp. (Korea). RPMI-1640 medium was obtained from Thermo Fisher Scientific (USA). Fetal bovine serum (FBS) and 1% penicillin/streptomycin were obtained from Welgene Inc. (Korea). EZ-Cytox cell viability analysis kit was obtained from Daeil Labservice (Korea). TRI reagent was purchased from Molecular Research Center, Inc. (USA). High-capacity cDNA reverse transcription kit was obtained from Thermo Fisher Scientific and TB Green Premix Ex Taq II was obtained from Takara Bio Inc. (Japan).

### Animals and Experimental Design

Male BALB/c mice (6 weeks old, 21−23 g) were obtained from Central Lab Animal Inc. (Korea). Before the experiment, these mice were acclimated for one week under the standard climate-controlled conditions. The Institutional Animal Care and Use Committee (IACUC) of Gangneung–Wonju National University, Korea, approved this work (Approval Number: GWNU-2018-20-2).

**Fig. 1:**
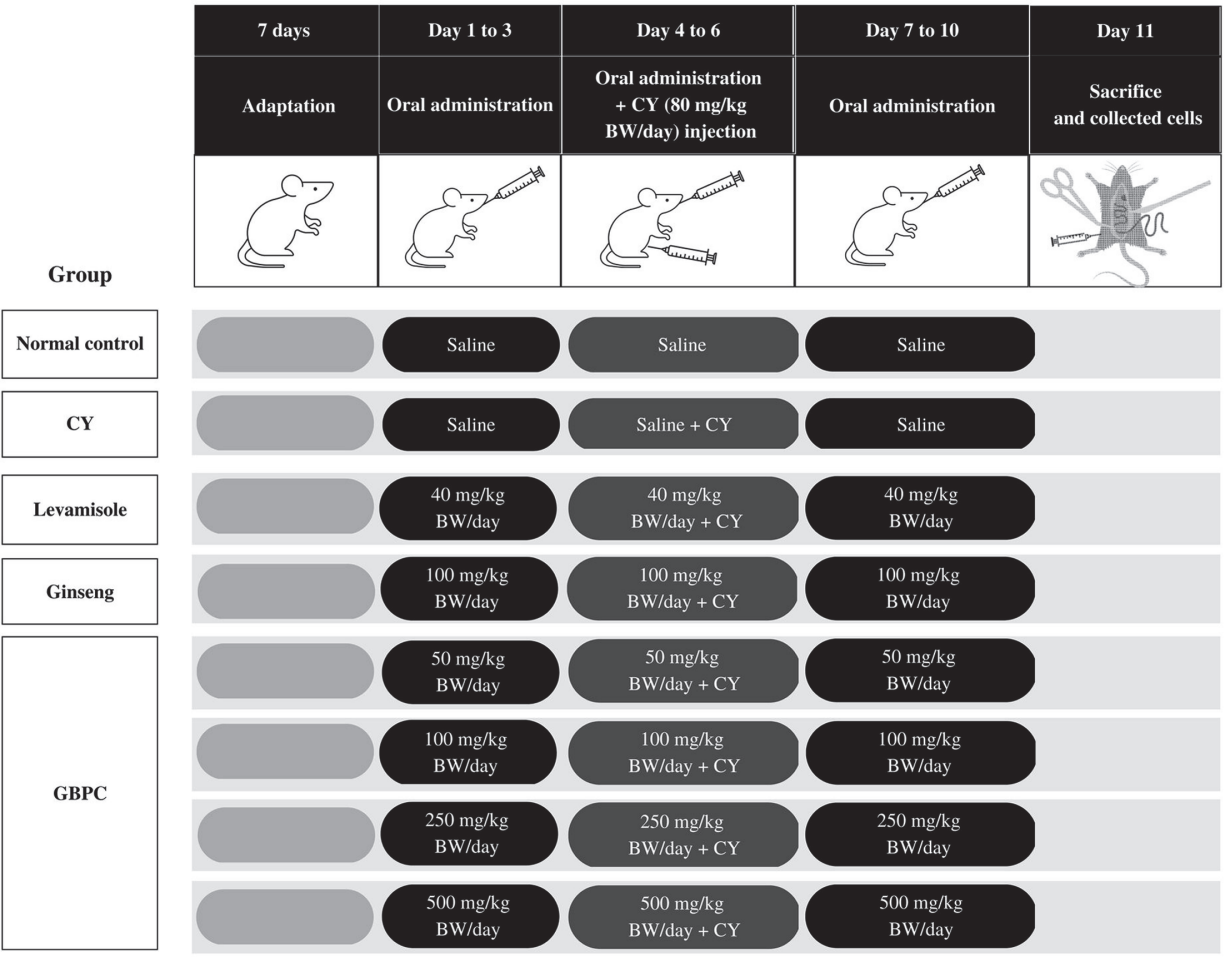
Scheme of animal experiment protocol. GBPC: crude polysaccharide extracted from Korean ginseng berry. BW: body weight. CY: cyclophosphamide. CY injection: mice were injected into the peritoneal cavity.

Fig. 1 shows the protocols for animal experiments and the establishment of immunosuppression mice. Following one week of adaptive breeding, mice were randomized into eight groups (*n* = 5 mice for each group). One group of healthy mice was employed as normal control (normal group), and treated every day with saline solution by gavage for 10 days. Other mice were orally administered every day for 10 days, as follows: CY group of mice as negative control were given saline solution; GBPC groups of mice were given different dosages of GBPC at 50, 100, 250, and 500 mg/kg BW/day; levamisole group of mice were given levamisole at 40 mg/kg BW/day; and ginseng group of mice were given commercial red ginseng syrup at 100 mg/kg BW/day. Positive controls included levamisole and commercially red ginseng syrup, which were immunostimulating agents of polysaccharides in immunosuppressed mice induced by CY [34, 35]. From days 4 to 6, all mice (excluding those in the normal group) were given an intraperitoneal injection of 80 mg/kg BW/day of CY to induce immunosuppression. Mice were weighed and sacrificed at 24 h after their last dosage.

### Preparation of Peritoneal Macrophages

Peritoneal macrophages were collected from the peritoneal cavity of each mouse after mouse was given an injection of 5 ml of 1× PBS buffer containing 3% FBS. The cell pellet of peritoneal macrophages was centrifuged at 400 ×*g* for 2 min, and washed with 1× PBS buffer twice. After that, RPMI-1640 medium containing with 1 %penicillin/streptomycin and 10% FBS was applied to the cell pellet. The cells of peritoneal macrophages were modified to have a density of 1 × 10^6^ cells/ml for further use.

### Determination of Nitric Oxide (NO) Production

Peritoneal macrophages (1 × 10^6^ cells/ml) were seeded into 96-well plates, and incubated for 1 h at 37°C in a humidified atmosphere of 5% CO_2_. Cells were removed, and activated by either with or without 1 μg/ml of LPS. After incubation for 24 h, nitrite accumulation in the culture solution was quantified to estimate the production of NO using Griess reagent. The culture supernatants (100 μl) were combined with 50 μl of Griess reagent A (1%sulfanilamide in 0.5% H_3_PO_4_) and 50 μl of Griess reagent B (0.1% *N*-1-naphthyl-ethylenediamine in distilled water). After 10 min at room temperature, the absorbance at 540 nm was evaluated that used a microplate reader (EL800; BioTek, USA).

### Determination of Peritoneal Macrophage Proliferation

An EZ-Cytox cell viability kit was used to assess the cytotoxicity of peritoneal macrophages. The cells (1 × 10^6^ cells/ml) in 96-well plates were activated by either with or without 1 μg/ml of LPS for 24 h. After incubation, the culture solution was removed, and the treated cells were added to each well with the 110 μl of diluted WST solution (WST: RPMI in the ratio of 1:10) for 1 h. A microplate reader was used to measure absorbance at 450 nm. The cell proliferation (%) was calculated as (the absorbance of the treated cells / the absorbance of the untreated cells) and setting the Normal group to 100 %.

### Phagocytosis Assay

Peritoneal macrophages (1 × 10^6^ cells/ml) were treated with or without LPS (6 μg/ml) for 24 h. The phagocytic ability of peritoneal macrophages was determined by a neutral red uptake assay [36]. Briefly, peritoneal macrophages were rinsed with 1× PBS buffer, added with 200 μl of 0.09% neutral red solution, and incubated at 37°C for another 30 min. After staining, cells were rinsed with 1× PBS buffer to eliminate excess neutral red. After adding with 100 μl of 50% ethanol containing 1% glacial acetic acid into each well. The absorbance was assessed using a microplate reader at 540 nm.

### Analysis of mRNA Expression by Quantitative RT-PCR

Peritoneal macrophages (1 × 10^6^ cells/ml) were placed into 24-well plates, and incubated for 1 h at 37°C. Cells were activated by either with or without 1 μg/ml of LPS, and incubated for 24 h. After incubation, TRI reagent was used to extract RNA from peritoneal macrophages. For cDNA synthesis, total RNA was reverse transcribed into cDNA using a High-capacity cDNA reverse transcription kit, as directed by the manufacturer. cDNA amplification was examined using TB Green Premix Ex Taq II and a QuantStudio 3 FlexReal-Time PCR System (Thermo Fisher Scientific, USA). The following primer sequences were utilized in real-time PCR: iNOS (forward: 5΄-TTCCAGAATCCCTGGACAAG-3΄ and reverse: 5΄-TGGTCAAACTCTTGGGGTTC-3΄); COX−2 (forward: 5΄-AGAAGGAAATGGCTGCAGAA-3΄, and reverse: 5΄-GCTCGGCTTCCAGTATTGAG-3΄); IL−1β (forward: 5΄-GGGCCTCAAAGGAAAGAATC-3΄ and reverse: 5΄-TACCAGTTGGGGAACTCTGC-3΄); IL−6 (forward: 5΄-AGTTGCCTTCTTGGGACTGA-3΄ and reverse: 5΄-CAGAATTGCCATTGCACAAC-3΄); TNF–α (forward: 5΄-ATGAGCACAGAAAGCATGATC-3΄ and reverse: 5΄-TACAGGCTTGTCACTCGAATT-3΄), and β-actin (forward: 5΄-CCACAGCTGAGAGGGAAATC-3΄ and reverse: 5΄-AAGGAAGGCTGGAAAGAGC-3΄).

### Statistical Analysis

Data are displayed as the mean ± standard deviation (SD). All statistical tests were analyzed using the Statistix 8.1 Statistics Software (USA) by one-way analysis of variance with Tukey post-hoc test. Statistically significant was considered when the *p* value was less than 0.05.

## Results

### Effects of GBPC on Peritoneal Macrophage Proliferation

**Fig. 2:**
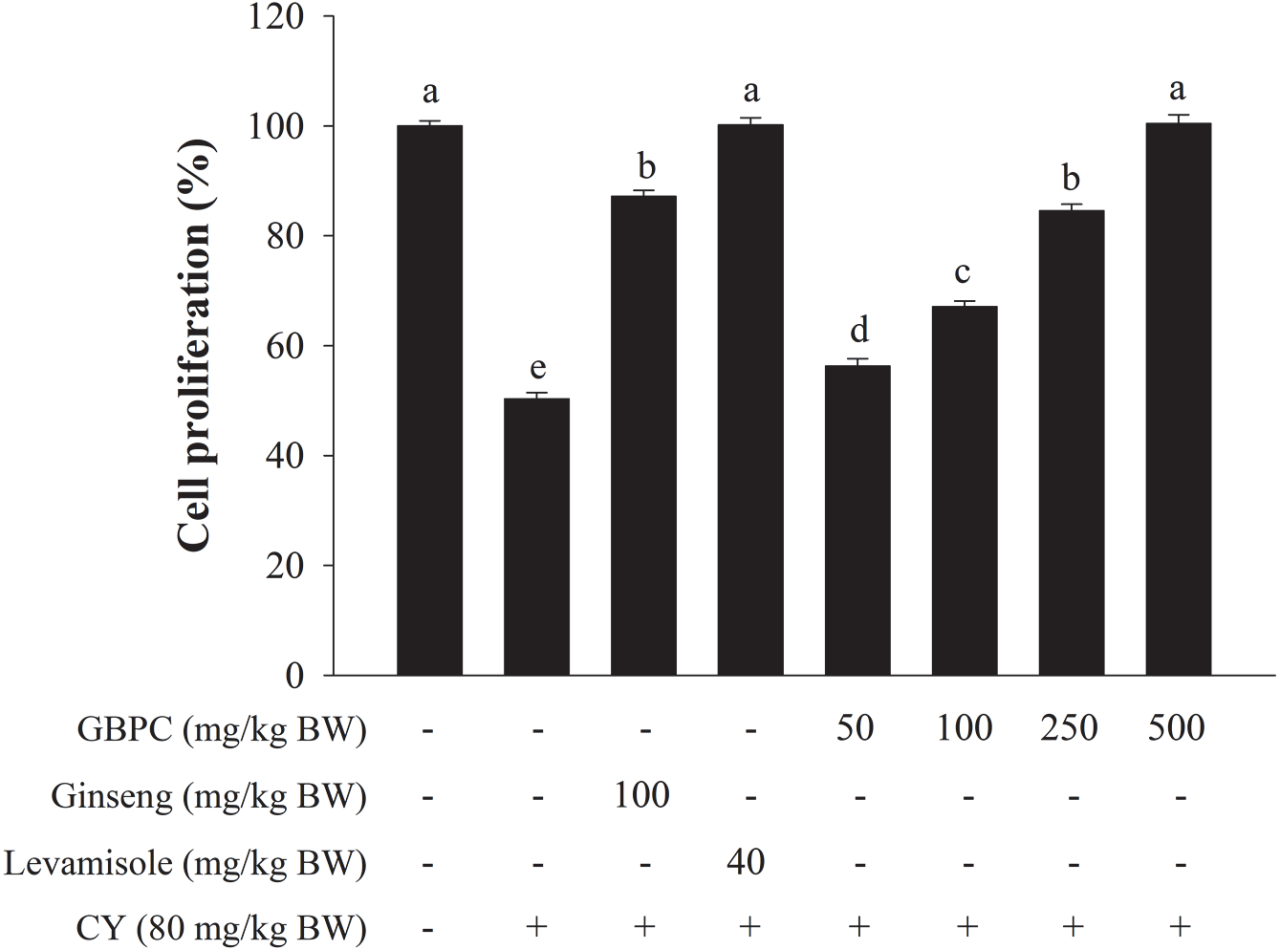
Effects of GBPC on peritoneal macrophage proliferation in CY-treated mice. Cells were placed into the 96- well plate at 1 × 10^6^ cells/ml with LPS (1 μg/ml). The cell proliferation was measured by WST method. Data are presented as the mean ± SD. A one-way ANOVA with a Tukey post-hoc test was performed for statistical analysis. Different letters (a, b, c, d, and e) indicate a significant difference (*p* < 0.05) between groups.

As shown in Fig. 2, 50−500 mg/kg BW/day of GBPC-treated groups promoted the cell proliferation in a dosage-dependent manner. GBPC of 50−500 mg/kg BW/day significantly improved by 56.3 − 100.4% compared to the CY group by 50.3%. In addition, the group administered with GBPC at 500 mg/kg BW/day and normal control or positive control group showed slightly different effects on the cell proliferation of peritoneal macrophages. The results indicate that levamisole, ginseng, and GBPC groups had no effect on macrophage viability in immunosuppressed mice.

### Effects of GBPC on the NO Production of Peritoneal Macrophages

**Fig. 3:**
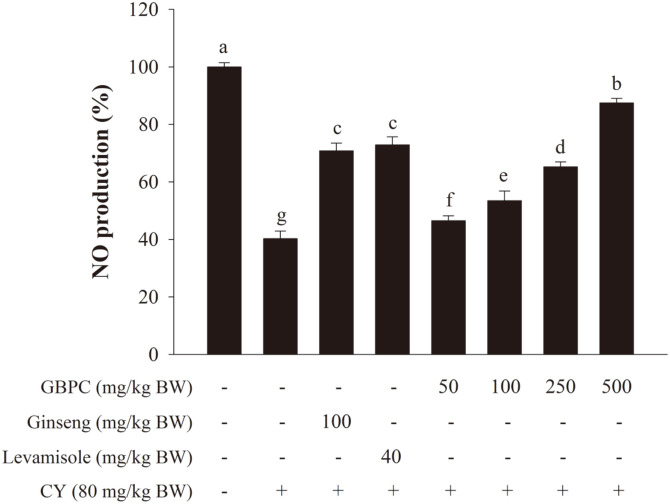
Effects of GBPC on NO production by peritoneal macrophages of CY-treated mice. Cells were placed into the 96-well plate at 1 × 10^6^ cells/ml with LPS (1 μg/ml). The nitrite accumulation was determined by Griess reagent. A one-way ANOVA with a Tukey post-hoc test was performed for statistical analysis. Data are presented as the mean ± SD. Different letters (a, b, c, d, e, f, and g) indicate a significant difference (*p* < 0.05) between groups.

To evaluate the immunomodulatory activity of GBPC on the production of NO in the immune system, mice were supplied with CY treatment as a test model. As shown in Fig. 3, all samples significantly reduced NO production, compared with the normal control (*p* < 0.05). Compared to CY-treated group at 40.3 ± 2.7%, the GBPC-treated groups at 50, 100, 250, and 500 mg/kg BW/day dosage-dependently increased NO production by 46.5 ± 1.7%, 53.5 ± 3.4%, 65.3 ± 1.7%, and 87.5 ± 1.6%, respectively. At a high dosage (500 mg/kg BW/day), the GBPC-treated group showed higher NO production than the positive control group, such as levamisole and ginseng at 72.9 ± 2.8% and 70.8 ± 2.7%, respectively.

### Effects of GBPC on the Phagocytic Activity of Peritoneal Macrophages

**Fig. 4:**
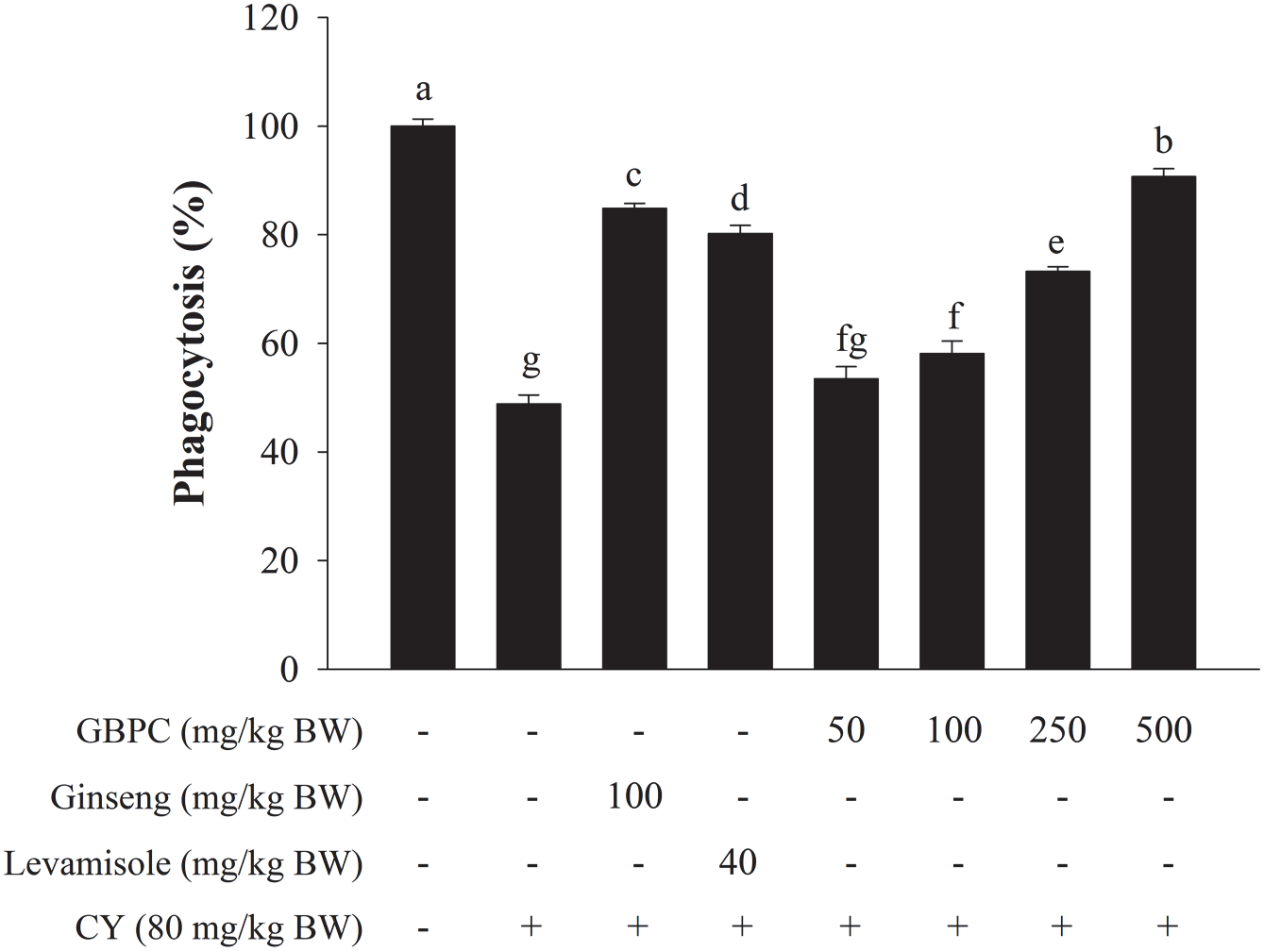
Effects of GBPC on the phagocytic activity of peritoneal macrophages in CY-treated mice. Cells were placed into the 96-well plate at 1 × 10^6^ cells/ml with LPS (6 μg/ml). The macrophage phagocytosis was determined by neutral red solution. Data are presented as the mean ± SD. A one-way ANOVA with a Tukey post-hoc test was performed for statistical analysis. Different letters (a, b, c, d, e, f, and g) indicate a significant difference (*p* < 0.05) between groups.

As shown in Fig. 4, the phagocytosis activity of peritoneal macrophages in CY-treated mice was considerably lower than in the normal group (100%). The phagocytosis activity was remarkably and dosage-dependently increased by GBPC at 53.5−90.7% of 50−500 mg/kg BW/day, compared with the CY group at 48.8 ± 1.7%. Compared with the CY group, the levamisole and ginseng groups also promoted the recovery of macrophage phagocytosis by 84.9 ± 0.9% and 80.2 ± 1.5%, respectively.

### Effects of GBPC on Immune-Related Gene Expression in Peritoneal Macrophages

**Fig. 5:**
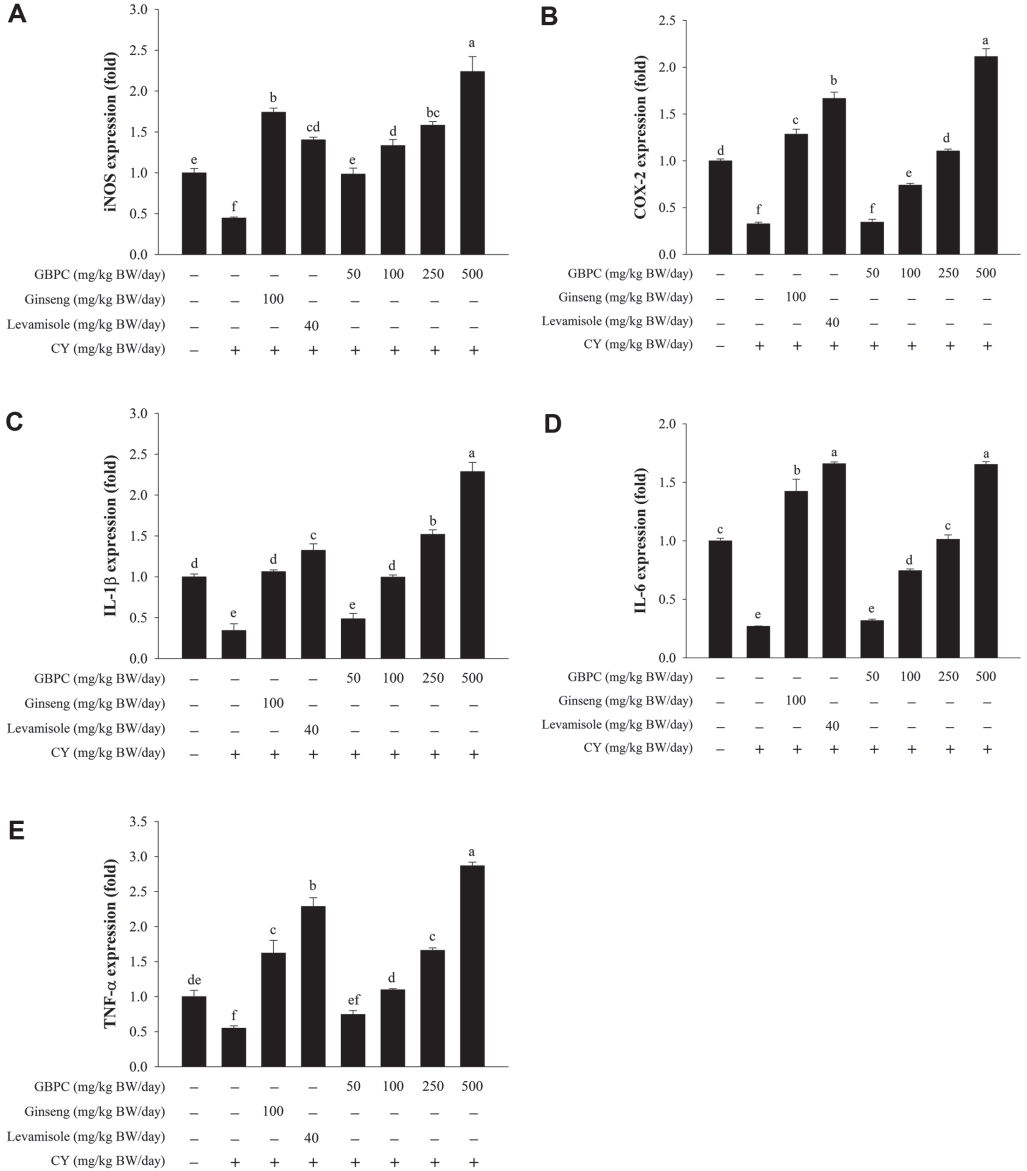
Effects of GBPC on the mRNA expression levels of cytokines in peritoneal macrophages of CY-treated mice. Expression levels of (**A**) *iNOS*, (**B**) *COX−2*, (**C**) *IL−1β*, (**D**) *IL−6*, and (**E**) *TNF–α* mRNA were determined by real-time PCR. Data are presented as the mean ± SD. A one-way ANOVA with a Tukey post-hoc test was performed for statistical analysis. Different letters (a, b, c, d, e, and f) indicate a significant difference (*p* < 0.05) between groups.

In this study, the mRNA expression levels of immune-associated genes in peritoneal macrophages of immunosuppressed mice were investigated. The results showed that the CY group had lower expression levels of immune-associated genes than the normal group. As shown in Figs. 5A and 5B, the mRNA expression levels of *iNOS* and *COX−2* were significantly enhanced by GBPC at 50−500 mg/kg BW/day in a dosage-dependent manner. Compared to the CY-treated group, the GBPC-treated group had a significant increase in the expression of pro-inflammatory cytokines *IL−1β*, *IL−6*, and *TNF–α* (Figs. 5C−5E). Furthermore, the expression levels of these genes in the GBPC (500 mg/kg BW/day) group were comparable to or greater than those in the levamisole and ginseng groups.

## Discussion

Polysaccharides isolated from Korean ginseng berries have been shown in vivo and in vitro to affect immune function via splenic lymphocytes and RAW 264.7 macrophages [32, 33]. In the present study, the immune-enhancing activities of GBPC on peritoneal macrophages in cyclophosphamide (CY)-induced mice were investigated. The immunomodulatory effect of CY has been studied in immunosuppressive animal models [11, 37, 38]. The CY alkylating agent is commonly used to treat cancer, but it is known for its serious side effects and widespread activity in harmful diseases like humoral antibody (HA), delayed-type hypersensitivity (DTH), and leukopenia, including oxidative stress [7]. At present, levamisole as a positive control is active against helminths, but it also enhances the immune system in normal, healthy laboratory animals [39], and has both immunostimulant and immunosuppressive properties, which contributed to regulating the immunological response caused by CY [34]. Additionally, ginseng was also used as a positive control, which has demonstrated the numerous pharmacological effects (anti-diabetic, anti-oxidative, anti-aging, and anti-tumor) and immunopotentiation on cellular immune function [22, 25, 40].

Among the different categories of immune cells (macrophages, splenocytes, NK cells, and others), macrophages have a crucial function in both the innate and adaptive immune systems by producing cytotoxicity and inflammatory chemicals, as well as secreting cytokines to fight external pathogens [1, 38]. Macrophage activation is a key defense mechanism against diseases and external invaders, and also serves as antigen-presenting cells and collaborate with T lymphocytes to regulate adaptive immunity [1, 13, 38]. The most common sources of macrophages are peritoneal cavity, spleen, and bone marrow [41]. In comparison to bone marrow-derived and splenic macrophages, peritoneal macrophages are significantly different from macrophages of other organs, express more inducible cytokines and have a more stable functional and phenotypic profile [41, 42]. Many previous studies found that most of the immunomodulators of the mouse peritoneal macrophages evaluated consisted of proliferation, pinocytic activity, NO levels, and cytokine secretion, and they affected the immune system in CY-treated mice of plant polysaccharides [11, 37, 38]. In the present study, GBPC significantly promoted macrophage proliferation in CY-treated mice, consistent with other studies reporting that plant polysaccharides can also enhance the cellular cytotoxicity [2, 18, 35]. Macrophages produce high amounts of NO to protect their host cells from infection [43]. NO is produced by nitric oxide synthase from L-arginine and molecular oxygen, a major effector molecule against pathogenic agents and tumor cells in non-specific immunity and immunological responses [11, 44]. Our results showed that GBPC stimulated macrophages to produce NO in immunosuppressive mice. Similar to our results, polysaccharides isolated from *Hippophae rhamnoides* berries can also significantly enhance the production of NO in peritoneal macrophages [2]. These findings imply that GBPC can enhance macrophage proliferation and NO generation.

Phagocytosis of macrophages is a key marker of pathogen microorganisms and is essential for the immunological responses of the body, including pathogen defense, tissue repair promotion, and chronic inflammation, and the phagocytic function of animal cells is commonly used to evaluate non-specific immunity [16, 37]. Administration of GBPC at 50−500 mg/kg BW/day) improved the ability of peritoneal macrophage phagocytosis. According to Yu *et al*. [14], American ginseng polysaccharides could raise immunological response by promoting macrophage phagocytosis at various concentrations of 50−200 μg/ml. Polysaccharides isolated from *Lycium barbarum* berries demonstrated the greatest immunological activity in terms of triggering phagocytosis and NO production in macrophage cells [45]. The current results indicate that GBPC could also improve the phagocytosis activities of peritoneal macrophages in immunosuppressive mice models.

Numerous multiple cytokines, which influence immunity cellular and humoral reactions, are produced by activated macrophages [1, 8]. Our previous study has found that GBPC and fractionated polysaccharides (F1, F2, and F3) from Korean ginseng berry can significantly upregulate the expression of *iNOS*, *IL−1β*, *IL−6*, and *TNF–α* in RAW 264.7 macrophages [32]. GBPC can also stimulate the expression of *IL−1β*, *IL−2*, *IL−4*, *IL−6*, *TNF–α*, *IFN–γ*, *TLR−4*, and *COX−2* in splenic lymphocytes [33]. In peritoneal macrophages, a mixture of Codium fragile polysaccharide and red ginseng extract can considerably increase the transcription levels of the genes for *TNF–α*, *IL−1β*, and *IL−6*, as well as *iNOS* and *COX−2* [35]. Glycosaminoglycan isolated from Apostichopus japonicus can also up-regulate IL−1β, IL−6, IL-18, MCP-1 and iNOS expression in peritoneal macrophages [10]. Our findings in this study revealed that GBPC stimulated *TNF–α*, *IL−1β*, *IL−6*, *COX−2*, and *iNOS* expression, indicating that GBPC could regulate inflammation through these cytokines. In addition, CY-treated mice given levamisole (at 40 mg/kg BW/day) or red ginseng (at 100 mg/kg BW/day) showed significantly enhanced immune function, compared to mice in the CY group. GBPC at 500 mg/kg BW/day resulted in obviously higher expression than levamisole and ginseng as positive controls of an immunostimulant agent [11, 35, 40].

## Conclusion

Our study demonstrated that GBPC exhibited potent immune-enhancing properties in the peritoneal macrophages of CY-induced immunosuppressive mice. GBPC treatment boosted NO generation and cell proliferation while enhancing the function of peritoneal macrophages in phagocytosis. Moreover, GBPC markedly up-regulated the mRNA expression of genes that contribute to immunity in immunosuppressive mice induced by CY. Consequently, these findings imply that GBPC may be used as an immunomodulatory agent under an immunosuppressive condition.
